# Environmental Impact of the Reclaimed Sand Addition to Molding Sand with Furan and Phenol-Formaldehyde Resin—A Comparison

**DOI:** 10.3390/ma13194395

**Published:** 2020-10-01

**Authors:** Mariusz Holtzer, Rafał Dańko, Angelika Kmita, Dariusz Drożyński, Michał Kubecki, Mateusz Skrzyński, Agnieszka Roczniak

**Affiliations:** 1 Faculty of Foundry Engineering, AGH University of Science and Technology, al. Mickiewicza 30, 30-059 Krakow, Poland; rd@agh.edu.pl (R.D.); dd@agh.edu.pl (D.D.); mskrzyns@agh.edu.pl (M.S.); arocznia@agh.edu.pl (A.R.); 2 Academic Centre for Materials and Nanotechnology, AGH University of Science and Technology, al. Mickiewicza 30, 30-059 Krakow, Poland; 3 Łukasiewicz Research Network—Institute for Ferrous Metallurgy, K. Miarki 12-14, 44-100 Gliwice, Poland; michal.kubecki@imz.pl

**Keywords:** metal casting, molding sand, emission, environmental protection, hazardous pollutants, phenol-formaldehyde resin, furan resin

## Abstract

Increasingly strict regulations, as well as an increased public awareness, are forcing industry, including the foundry industry, to develop new binders for molding sands, which, while being more environmentally friendly, would simultaneously ensure a high quality of castings. Until recently, binders based on synthetic resins were considered to be such binders. However, more accurate investigations indicated that such molding sands subjected to high temperatures of liquid metal generated several harmful, even dangerous substances (carcinogenic and/or mutagenic) from the benzene, toluene, ethylbenzene and xylenes (BTEX) and polycyclic aromatic hydrocarbons groups (PAHs). An assessment of the most widely used molding sands technologies at present with organic binders (synthetic resins) from the no-bake group (furan no-bake and phenolic-ester no-bake) and their harmfulness to the environment and work conditions is presented in this paper. In the first stage of this research, gases (from the BTEX and PAHs groups) emitted when the tested molds were poured with liquid cast iron at 1350 °C were measured (according to the authors’ own method). The second stage consisted of measuring the emission of gases released by binders subjected to pyrolysis (the so-called flash pyrolysis), which simulated the effects occurring on the boundary: liquid metal/molding sand. The gases emitted from the tested binders indicated that, in both cases, the emission of harmful and dangerous substances (e.g., benzene) occurs, but, of the given binder systems, this emission was lower for the phenolic-ester no-bake binder. The obtained emission factors of BTEX substances show higher values for furan resin compared to formaldehyde resin; for example, the concentration of benzene per 1 kg of binder for furan no-bake (FNB) was 40,158 mg, while, for phenol-formaldehyde no-bake (PFNB), it was much lower, 30,911 mg. Thus, this system was more environmentally friendly.

## 1. Introduction

Metal casting involves pouring a molten metal into a hollow mold to produce metal objects. Cores are used in a casting process to form cavities, hole protrusions, recesses and casting products, which are not possible to be shaped by the mold. These molds and cores are generally made of molding sands and are chemically bound, bentonite clay, or unbound [1,2]. Some of the chemical compounds that are used are phenolic-urethane, furan and phenolic-formaldehyde resins. When a mold or core is in contact with liquid metal, as a result of a heat influence, thermo-mechanical and thermo-chemical processes occur, causing dimensional changes at the mold and/or core boundary, which negatively influence the casting quality.

The foundry industry is developing at an extremely fast pace and consumes large amounts of natural resources, energy and metals, generating significant amounts of gases and solid wastes, which influence the natural environment and work conditions. Thus, foundries are implementing more environmentally friendly, energy-saving and efficient molding forming methods. Significant amounts of molds and cores are produced from chemically bound molding sands, in which binders are based on synthetic resins. Chemically bound sands have some better molding sand parameters than green sands [2,3,4,5]. Heat activated, no-bake and cold-box are three main classes of organic binder systems. The following resins are applied in practice in foundries: alkyd, phenol-formaldehyde, furan and urea-formaldehyde. These resins, under the influence of high temperatures of liquid metals, are subjected to degradation, resulting in the emission of hazardous substances that are deadly to humans [6,7,8,9,10,11]. However, because trace metals may accumulate in molding sands during casting, there is a concern over the contamination of terrestrial and aquatic environments [12,13].

Depending on the foundry, the sand can be reclaimed and used to make molds and cores. The waste sands are then often disposed of in landfills. However, this disposal in landfills is becoming an increasing problem as legislation is getting tighter and the disposal cost by current practices increases rapidly [14,15,16,17,18]. Among the 187 Hazardous Air Pollutants (HAPs), some 40 compounds have been identified in the air emissions from foundries [19]. These HAPs are released during metal pouring, mold cooling and casting shakeout when the carbonaceous additives are exposed to the casting heat. As numerous investigations indicate, including those of the authors [9,11,20], the formed substances, of which several are dangerous, e.g., BTEX and PAHs, are partially condensed on matrix grains and together with molding sand undergo the mechanical reclamation process [21,22,23,24]. The dominate molding sand technologies with chemically-bound binders are furan no-bake, phenolic-urethane no-bake and ester-cured-phenolic no-bake. Furan-based binders are currently the most widely used organic binders in the metal casting industry, accounting for almost half of all organic substances consumed in foundries worldwide [25]. However, in the U.S., the phenolic-urethane no-bake (PUNB) technology is widely applied (nearly 25% in the U.S vs. only 3% in the UE). In the field of cores production technologies, the phenolic-urethane cold-box (PUCB) process is dominant in both geographic regions (more than 30%).

The following elements should be considered when selecting the molds and cores production technology: yield, casting quality and influence on the environment and work conditions, which in consequence decides the costs.

Advantages of the no-bake technology are low resin addition, high reactivity, low viscosity, high strength and good reclaimability of the sands [26].

Their drawbacks constitute problems with emissions of formaldehyde, phenol, benzene and their derivatives, as well as sulfur compounds during production and storing of molds and cores and pouring them with liquid metal; high prices of furfuryl alcohol (monopoly of China); short service-life of sand; and nitrogen content in furfuryl resins (disadvantageous for steel castings).

According to the principle of “zero wastes”, sands from both technologies after knocking out are subjected to the reclamation process, which is the removal of hardened binders from surfaces of sand grains [22,23]. In the case of the mechanical reclamation, this removal is not complete. When the so-reclaimed sand substitutes fresh sand, the remaining binders under the influence of liquid metal high temperature undergo destruction and additional gases are emitted.

There are two primary sources of emissions from resin-based binders:

- Evaporation of solvent, byproduct or chemical constituent occurring during mixing, core/mold making and core/mold storage, prior to pouring
- Thermal decomposition during pouring, cooling and shakeout operations.

Special attention should be placed on the emission of substances from the BTEX and PAHs groups since many of them are carcinogenic and mutagenic. The aim of these study was not only the determination of the emission of substances in the BTEX and PAHs groups from the molding sands with furfuryl and phenolic-formaldehyde resins under the laboratory scale but also determining the influence of the reclaimed sand addition. All these elements are factored into the overall assessment of the harmful influence of the given molding sand on the environment and employees. Due to this, it is possible to protect nature against hazardous substances [27,28,29,30].

## 2. Materials and Methods

### 2.1. Materials 

Molding sands of no-bake type were tested:

- Furan no-bake acid catalyzed (FNB) was composed of 1% urea-furfuryl resin (free furfuryl alcohol 85%; nitrogen <0.9%; free formaldehyde <0.1%); hardener (p-toluenesulfonic acid) and fresh silica sand or reclaimed sand.
- Phenolic esters no-bake (PFNB) was composed of 1.2% phenol-formaldehyde resin (free phenol, 1.0%; free formaldehyde <0.20%); hardener (mix of organic esters) and fresh silica sand or reclaimed sand.

Table 1 shows the chemical composition of the silica sands. Table 2 gives the grain size distribution of the fresh and reclaimed silica sand.

### 2.2. Methods

**Figure 1:**
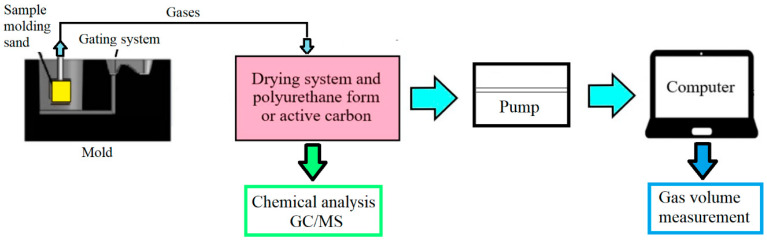
Scheme of the stand for measuring the gas volume and BTEX and PAHs emission. A detailed description of the methodology and apparatus can be found in [29].

Investigations of the gas emission in the tested foundry plant were performed according to the original method developed in the Faculty of Foundry Engineering, AGH-UST (Polish Patent, No. P-398 709; 2012). The schematic presentation of the experimental stand is given in Figure 1.

#### 2.2.1. Gas Chromatography/Mass Spectrometry (GC/MS) 

The analysis was carried out using Gas Chromatograph (GC) Trace 1310 (Thermo Scientific, Waltham, MA, USA) with TriPlus RSH autosampler (Thermo Scientific, Waltham, MA, USA) with PTV (Thermo Scientific, Waltham, MA, USA), S/SL dispenser (Thermo Scientific, Waltham, MA, USA) and Headspace (Thermo Scientific, Waltham, MA, USA), coupled with Mass Spectrometer (MS) MS 1100 ion trap type (Thermo Scientific, Waltham, MA, USA). Gas products were separated on chromatographic columns:

- BTEX analysis: HP-5MS; 30 m × 0.25 mm × 0.25 μm (film thickness) capillary column
- PAHs analysis: ZB-PAH; 20 m × 0.18 mm × 0.14 μm (film thickness) capillary column

Mass Spectrometer (MS) was used to detect the degradation products:

- BTEX analysis (source temperature: 250 °C; operating mode: SIM)
- PAHs analysis (source temperature: 250 °C; operating mode: SCAN)

#### 2.2.2. Pyrolysis-Gas Chromatography/Mass Spectrometry (Py-GC/MS) 

The analysis was carried out in a platinum coil. Approximately 1 mg of the solid sample was centered in a quartz tube and heated up to 1100 °C (heating ramp of 10 °C/ms) using a (Py) pyroprobe (Pyroprobe 5000, CDS, Analytical Inc., Oxford, PA, USA). The pyrolysis products were separated using Gas Chromatograph on a 30 m × 0.25 mm × 0.25 μm (film thickness) capillary column (Rxi-5MS, Restek, Bellefonte, PA, USA). The flow rate of the carrier gas (He, 99.9999%) was 1 mL/min. A Single Quadrupole (ISQ, Thermo Scientific, Waltham, MA, USA) MS was used to detect the pyrolytic degradation products (scan mode: (30–600) atomic mass units (a.m.u.); electron energy (EI): 70 eV; emission current: 50 μA). The obtained mass spectra were compared with the mass spectra given in the NIST MS Search 2.0 Libera (Chemm. SW, Version 2.0, Fairfield, CA, USA).

- During the first stage of the research, the composition of gases evolving from molding sands, prepared on fresh sand matrices and poured with cast iron with a temperature of 1350 °C, was tested (samples marked: FNB and PFNB) [11].
- During the second stage, the emission of substances evolving from molding sands, prepared on fresh sand matrices with various fractions of a reclaim and poured with cast iron of a temperature of 1350 °C, was measured (samples marked: FNBXRYFS, where XR is the percent of reclaim fraction and YFS is the percent of fresh sand fraction).
- During the third stage, the “flash pyrolysis” of hardened resins was performed by Pyrolysis–Gas Chromatography–Mass Spectrometry (Py-GC/MS) technique at a temperature of 1100 °C. This experiment simulated processes occurring directly on the boundary of molding sand and liquid alloy [13,30].

To determine the influence of reclaimed sand added to matrices of tested molding sands on the amount and kind of emitted gases, molding sands containing 100%, 50% and 0% of reclaimed sand were prepared and marked as follows:

- Molding sand with furan resin: FNB100FS (100% fresh sand), FNB50R50FS (50% reclaimed sand + 50% fresh sand) and FNB100R (100% reclaimed sand).
- Molding sand with phenol-formaldehyde resin: PFNB100FS (100% fresh sand), PFNB50R50FS (50% reclaimed sand + 50% fresh sand) and PFNB100R (100% reclaimed sand).

All samples were subjected to the same research procedure. The analysis of BTEX was carried out using the gas chromatography method with the application of a flame-ionizing detector (FID) (TRACE GC Ultra Thermo Scientific, Waltham, MA, USA).

Substances from the PAHs group were analyzed using the gas chromatography technique (FOCUS GC) coupled with MS ISQ Thermo Scientific (GC/MS, Waltham, MA, USA).

## 3. Results and Discussion

### 3.1. Investigations of the Gases Emitted from Molding Sands Prepared on the Fresh Sand Matrix

The obtained results of the research (Table 3 and Table 4) conducted according to the methodology developed by the authors [29] showed that for both BTEX and PAHs emission from the FNB molding sand is greater than from the PFNB molding sand. This is consistent with the research presented in [10]. The results of analyses of gases from the BTEX group emitted from FNB and PFNB molding sands, prepared on the fresh sand matrix, are shown in Table 3. The total volume of gases emitted from the PFNB molding sand was more than 20% greater than from FNB molding sand.

Among the BTEX gases emitted from both molding sands, benzene was predominant, constituting more than 90%. Moreover, despite the fact that the volume of gases emitted from the FPNB sand was higher than from FNB sand, emission of BTEX, including carcinogenic benzene, recalculated for 1 kg of molding sand, was lower by approximately 25%. The process of gases emission reached the maximum speed after 100 s, while the emission ended after approximately 250 s from pouring liquid metal into the mold.

Both tested molding sands under a high temperature influence also emit substances from the PAHs group (Table 4). Emissivity of these substances from FNB molding sand is approximately 20% higher than from PFNB molding sand. The main PAHs compounds emitted from FNB sand are fluoranthene, pyrene and phenanthren, while from PFNB sand they are pyrene and acenaphtylene. The concentration of benzo(a)pyrene, a highly carcinogenic substance, is very low for both binders.

**Figure 2:**
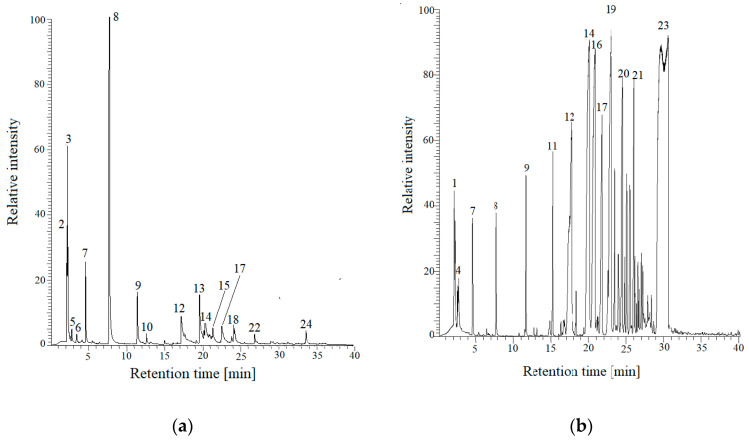
(**a**) Chromatogram obtained for the sample of furan resin (FNB) binder at the temperature of 1100 °C; and (**b**) chromatogram obtained for the sample of phenol-formaldehyde (PFNB) resin binder at the temperature of 1100 °C [31].

“Flash” pyrolysis simulated the pouring temperature of casting alloys (from nonferrous alloys through cast iron to cast steel). Investigation of compounds formed during the “flash” pyrolysis was conducted by means of the coupled equipment consisting of Py-GC/MS. Pyrolysis was carried out at 1100 °C. Analytical data collecting information from the Py-GC/MS techniques can be found in Table 5. Figure 2a,b shows chromatograms obtained for FNB and PFNB, respectively, at temperature of 1100 °C.

The chromatograms obtained for both resins are very similar in terms of qualitative analysis (type of evolved gases), while differences occur in the concentration range. In both cases, mainly gases from the BTEX group and their derivatives (one, two and three methylene) and phenol with its derivatives are emitted. Gases released from FNB resin contain 50% toluene, while it is negligible in gases emitted by the PFNB resin. Gases released from the FNB resin are also present: SO_2_ originated from the p-toluenesulfonic acid (PTSA) (hardener) and nitrogen compounds (probably introduced at the resin production stage).

### 3.2. Investigations of the Gases Emitted from Molding Sands Prepared on the Reclaimed Sand Matrices

**Figure 3:**
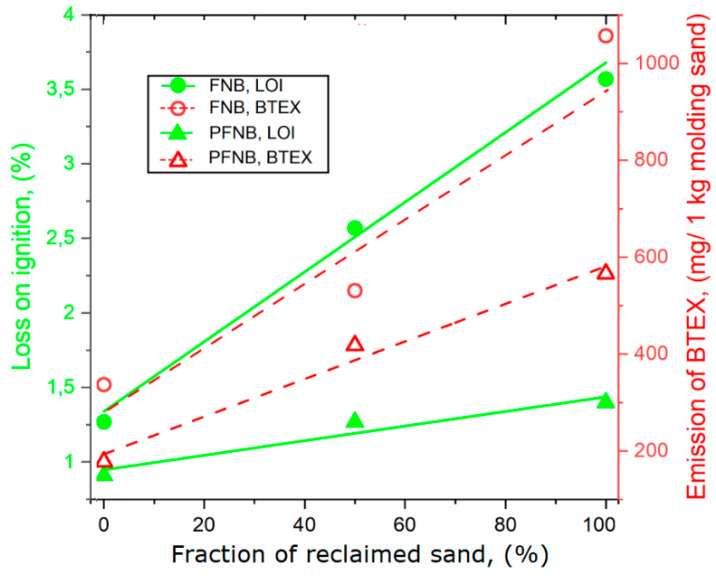
The dependence of emission of BTEX on the LOI of the given molding sand with reclaimed sand fraction [32,33].

An addition of the reclaimed sands to matrices of both molding sands caused a significant increase in the volume of emitted gases. For the FNB sand, this volume doubled (when the matrix was made of 100% of reclaimed sand), while for the PFNB sand the increase was lower (70%) (Table 2 and Table 6). The mechanical reclamation process of spent molding sand does not fully remove the hardened binder from sand grains. Thus, by adding the reclaimed sand to sand matrices, additional amounts of inactive binder are introduced into molding sands, increasing the volume of emitted gases and constituting a higher risk to the environment. The parameter LOI can be a measure of the binder remaining on the reclaimed sand grains. The dependence of the LOI of the given molding sand on the reclaimed sand fraction in its matrix is presented in Figure 3. Both the BTEX emission and the LOI values are proportional to the fraction of reclaimed sand (Figure 3). The LOI for the tested molding sand should be ≤2; this level of organic substances can be obtained by thermal reclamation [23]. However, high costs of energy carriers lead foundries to mainly apply mechanical reclamation, which is less efficient. Not totally removing binders from sand grain surfaces can be the reason for casting defects, due to emission of too high amounts of gases and environmental contamination.

The results of emission of substances from the BTEX group from molding sands having matrices with the reclaimed sand fraction 0%, 50% and 100% under a high temperature influence are shown in Table 6. Total substitution of fresh sands by reclaimed sands in FNB molding sand caused a threefold increase in the concentration of BTEX substances (fresh sand, 336.6 mg per 1 kg molding sand; 100% reclaimed sand fraction, 1057 mg per 1 kg molding sand).

As far as substances from the PAHs group are concerned, PFNB molding sand also releases less of these substances than FNB. The amounts of PAHs emited from PFNB molding sand are at the same level, regardless of the reclaimed sand fraction in a molding sand (Table 7).

However, for the FNB molding sand, the amount of emitted PAHs significantly increases when the reclaimed sand fraction in the matrix increases. When the matrix contained 100% reclaimed sand, the emission of substances from the PAHs group was double that when the matrix contained only fresh sand. The main component of released PAHs was naphthalene, which in a critical case constituted nearly 70% all PAHs. Due to the carcinogenic and/or mutagenic influence of substances occurring in these gases (benzene, toluene, formaldehyde and benzo(a)piren), which are either the initial components or products of reactions occurring under the high temperature influence in tested binders, the intensive research leading to the elimination or limitation of such components is being carried out, e.g. phenol free, formaldehyde free, furfuryl alcohol free and sulfuric acid free.

The results presented in this paper only concern the tested binder systems and should not be generalized. Investigations of new systems are necessary to properly assess their influence on the environment.

## 4. Conclusions

An assessment of the harmfulness for the natural and work environment created by gases from BTEX and PAHs groups released at high temperature from two molding sands with organic resin binders was performed. The most widely applied binders in the production of molds are furan no-bake acid catalyzed (FNB) and phenolic esters no-bake (PFNB). The following conclusions can be drawn:

- Molding sands with the PFNB binder release a low amount of substances from the BTEX group (by up to 25%) than molding sands with the FNB binder. In both cases, benzene constituted more than 90%.
- Molding sands with the PFNB binder release nearly 50% less substances from the PAHs group than molding sands with the FNB binder.
- The fresh sand substitution by a reclaimed sand in the matrix causes a significant increase (up to threefold) of the released gases from both groups. In addition, BTEX emission from the PFNB binder is twice lower than that from the FNB binder. The reclaimed sand addition to the PFNB sand influences the emission of PAHs only to a small degree.
- The LOI parameter can be useful in the case of these molding sands—for assessing amounts and approximated composition (e.g., benzene content) of evolving gases.

## Figures and Tables

**Table 1:** Chemical composition of silica sand.

**Component**	SiO_2_	Al_2_O_3_	Fe_2_O_3_	TiO_2_	CaO	MgO
**Mass (%)**	98.10	0.65	1.07	0.01	0.10	0.07

**Table 2:** Grain size distribution of fresh and reclaimed silica sand.

Grain Size (mm)	1.00	0.800	0.630	0.400	0.320	0.200	0.160	0.100	0.071	0.560	Total
**Fresh Silica Sand**											
**Fraction (%** **)**	0.00	2.50	6.68	23.95	23.17	34.15	6.23	3.29	0.00	0.00	100
**Reclaimed Silica Sand**											
**Fraction (%)**	0.02	1.15	5.13	34.04	25.88	25.86	5.51	2.16	0.05	0.00	100

**Table 3:** Concentration of BTEX formed during thermal decomposition of molding sands FNB and PFNB.

Sam-ple	Volume of Gases per 1 kg		Benzene per 1 kg		Toluene per 1 kg		Ethylbenzene per 1 kg		Xylenes per 1 kg		Total per 1 kg	
-	of molding sand, dm^3^	of binder,dm^3^	of molding sand, mg	of binder, mg	of molding sand, mg	of binder, mg	of molding sand, mg	of binder, mg	of molding sand, mg	of binder, mg	of molding sand, mg	of binder, mg
**Molding Sand with Furan Resin (85% of Free Furfuryl Alcohol) (FNB)**												
FNB	14.466	964	602	40,158	51	3384	0.77	51	4	260	658 ± 66	43,835 ± 4385
**Molding Sand with Phenol Formaldehyde Resin (PFNB)**												
PFNB	17.482	1161	464	30,911	26	1738	0.84	56	4	289	495 ± 50	32,994 ± 3299

**Table 4:** Quantity of PAHs compounds released from the FNB and PFNB molding sands during the thermal decomposition (fresh sand).

Compound	FNB Sand Molding		PFNB Sand Molding	
	Results per 1 kg of Binder, mg	Results per 1 kg of Molding Sand, mg	Results per 1 kg of Binder, mg	Results per 1 kg of Molding Sand, mg
Naphtalene	93	1.39	193	2.89
Acenaphtylene	26	0.40	128	1.92
Acenaphtene	2	0.03	2	0.02
Fluorene	9	0.14	15	0.23
Phenanthrene	102	1.52	33	0.50
Anthracene	59	0.88	8	0.15
Fluoranthene	176	2.63	66	1.00
Pyrene	129	1.92	64	0.96
Benz(a)anthracene	22	0.32	15	0.22
Chrysene	29	0.43	19	0.28
Benzo(b)fluoranthene	17	0.26	9	0.13
Benzo(k)fluoranthene	10	0.15	5	0.08
Benzo(a)pyrene	16	0.24	11	0.17
Indeno[1,2,3-cd]pyrene	55	0.83	34	0.51
Dibenz[a,h]anthracene	24	0.36	20	0.30
Benzo[g,h,i]perylene	40	0.60	34	0.51
Total PAHs ± 20%	806 ± 161	12.09 ± 2.42	658 ± 132	9.87 ± 2

**Table 5:** Identified pyrolysis products of cured resin FNB and PFNB at 1100 °C.

Peak Number	Retention Time (min)	CAS Registry Number	Compound Name/ Summary Formula	Molecular Weight g/mol	Relative Area *(%)	
					FNB Resin	PFNB Resin [31]
1	2.12	75–31–0	2-Propanamine, C_3_H_9_N	59	-	0.59
2	2.14	140308	Benzeneethanamine, 3-fluoro-beta,5-dihydroxy-N-methyl-, C_9_H_12_FNO_2_	185	1.43	-
3	2.20	7446–09–5	Sulfur dioxide-, SO_2_	64	10.78	-
4	2.23	67–56–1	Methanol, CH_4_O	32	-	0.27
5	2.77	646–05–9	1-Penten-3-yne C_5_H_6_	66	0.87	-
6	3.39	930–27–8	Furan, 3-methyl-, C_5_H_6_O	82	1.22	-
7	4.61	71–43–2	Benzene, C_6_H_6_	78	6.38	0.75
8	7.77	108–88–3	Toluene, C_7_H_8_	92	48.63	0.98
9	11.65	108–38–3	Benzene, 1,3-dimethyl-, C_8_H_10_	106	5.91	1.45
10	12.68	108–38–3	m-Xylene, C_8_H_10_	106	1.17	-
11	15.23	526–73–8	Benzene, 1,2,3-trimethyl-, C_9_H_12_	120	-	1.98
12	17.62	108–95–2	Phenol, C_6_H_6_O	94	3.24	2.95
13	20.47	108–40–7	Benzenethiol, 3-methyl-, C_7_H_8_S	124	3.24	-
14	20.65	106–44–5	m-Cresol, C_7_H_8_O	108	5.56	61.74
15	21.48	576–26–1	Phenol, 2,6-dimethyl-, C_8_H_10_O	122	1.69	-
16	21.68	95–87–4	Phenol, 2,5-dimethyl-, C_8_H_10_O	122	-	4.28
17	22.64	105–67–9	Phenol, 2,4-dimethyl-, C_8_H_10_O	122	3.57	14.35
18	24.28	28715–26–6	Benzofuran, 4,7-dimethyl-, C_10_H_10_O	146	2.90	
19	24.43	2416–94–6	Phenol, 2,3,6-trimethyl-, C_9_H_12_O	136	-	9.44
20	26.19	627–93–0 ^●^	Hexanedioic acid, dimethyl ester, C_8_H_14_O_4_	174	-	-
21	26.56	2219–78–5	2-methyl-4,5-dimethylphenol, C_10_H_14_O	150	-	1.22
22	27.02	1904–26–4	3-Buten-2-one, 3-methyl-4-phenyl-, C_11_H_12_O	160	1.85	-
23	29.54	102–62–5 ^●^	Glycerol 1,2-diacetate, C7H_12_O_5_	176	-	-
24	33.83	620–47–3	1-benzyl-3-methylbenzene, C_14_H_14_	182	2.78	-

● These compounds were not included in the quantification of degradation products. * Relative areas of the peaks indicative of the released products, calculated as the percent area = area of the compound divided by the total area of all of the integrated compounds.

**Table 6:** Concentration of BTEX formed during thermal decomposition of molding sand (FNB and PFNB) with reclaimed sands [32,33].

Sample Code	Volume of Gases per 1 kg of Molding Sand, dm^3^	Emission of Gases, mg/1 kg Molding Sand					Loss on Ignition LOI %
		Benzene	Toluene	Ethylbenzene	Xylenes	Total BTEX	
**Molding Sand FNB (Free Furfuryl Alcohol 50%)**							
FNB100FS	12.804	333	3	0.6	0	336.6	1.27
FNB50R50FS	18.261	513	18	0	0	531	2.57
FNB100R	24.369	957	91	1	8	1057	3.57
**Molding Sand PFNB**								
PFNB100FS	10.606	175	2	0.6	0	177.5	0.91
PFNB50R50FS	13.199	407	9.5	0.8	0.7	418	1.27
PFNB100R	17.568	553	11	1.2	0.8	566	1.40

**Table 7:** Quantity of PAHs released from FNB and PFNB during thermal decomposition of molding sand with reclaimed sands [32,33].

Compound	Results per 1 kg of Molding Sand, mg					
	Molding Sand PFNB			Molding Sand FNB		
	PFNB100FS	PFNB50R50FS	PFNB100R	FNB100FS	FNB50R50F	FNB100R
Naphtalene	1.66	2.18	2.02	3.12	5.12	10.18
Acenaphtylene	0.00	0.00	--	--	0.00	0.01
Fluorene	0.02	0.07	0.03	0.08	0.10	0.90
Phenanthrene	0.17	0.45	0.40	0.37	0.46	0.85
Anthracene	0.09	0.10	0.26	0.14	0.17	0.32
Fluoranthene	0.60	0.57	0.76	0.84	1.84	0.95
Pyrene	0.60	0.59	0.68	0.59	1.48	0.71
Benz(a)anthracene	0.20	0.23	0.10	0.26	0.40	0.17
Chrysene	0.54	0.73	0.08	0.14	0.38	0.09
Benzo(b)fluoranthene	0.37	0.36	0.28	0.49	0.75	0.18
Benzo(k)fluoranthene	0.14	0.15	0.08	0.19	0.25	0.07
Benzo(a)pyrene	0.48	0.48	0.33	0.80	1.04	0.23
Dibenz[a.h]anthracene	--	--	0.00	0.08	0.02	0.02
Benzo[g.h.i]perylene	0.36	0.48	0.30	0.36	0.05	0.13
Indeno[1.2.3 cd]pyrene	0.05	0.49	0.36	0.48	0.83	0.16
Total PAHs ± 20%	5.28 ± 1.06	6.88 ± 1.38	5.68 ± 1.14	7.94 ± 1.59	12.89 ± 2.58	14.97 ± 2.99
